# Structural validity of the Brazilian version of the Sense of Coherence scale (SOC-13) in oral health research: exploratory and confirmatory factor analysis

**DOI:** 10.1186/s12903-022-02373-1

**Published:** 2022-08-09

**Authors:** Roger Keller Celeste, Giovana Pereira Scalco, Claides Abegg, Marcos Pascoal Pattussi, Helenita Correa Ely, Rosane Silvia Davoglio, Maria do Carmo Matias Freire

**Affiliations:** 1 Departamento de Odontologia Preventiva e Social, Universidade Federal do Rio Grande do Sul, Rua Ramiro Barcelos 2492, 3º andar, Porto Alegre, RS 90035-003 Brazil; 2 School of Health and Life Sciences, Pontifical Catholic University of Rio Grande do Sul, Porto Alegre, Brazil; 3 Graduate Program in Collective Health, Universidade do Vale do Rio dos Sinos, São Leopoldo, Brazil; 4 Special Coordination of Biosciences and One Health, Federal University of Santa Catarina, Curitibanos, Brazil; 5 Graduate Program in Dentistry, Federal University of Goiás, Goiania, Brazil

**Keywords:** Sense of Coherence, Orientation to Life Questionnaire, Factor analysis, Confirmatory factor analysis, Oral health, Psychometric, SOC-13

## Abstract

**Background:**

The Sense of Coherence (SOC) construct has been used worldwide in oral health research, but rigorous factor analyses of the scale are scarce. We aim to test the dimensional structure of the Brazilian short version of the SOC scale with 13 items.

**Methods:**

This study is a secondary analysis of four independent cross-sectional Brazilian studies on oral health, using the 13-items SOC scale. Sample 1 was conducted on 1760 mothers and 1771 adolescents. Sample 2 comprised 1100 adults. Sample 3 had 720 adults and older individuals. Sample 4 comprised 664 adolescent students. Confirmatory Factor Analysis (CFA) was conducted on sample 1 to compare two models: 3-factor versus 1-factor. Because they were refuted, Exploratory Factor Analysis was implemented in samples 2 and 3. Modified models were tested in sample 4 using CFA. All analyses were conducted with MPlus version 7.11.

**Results:**

CFA of sample 1 resulted in an unacceptable fit (RMSEA = 0.12;CFI = 0.78; TLI = 0.73; and WRMR = 3.28) for 1-factor model and 3-factor (RMSEA = 0.10; CFI = 0.87; TLI = 0.84; and WRMR = 2.50). The EFA on samples 2 and 3 showed, respectively, two eigenvalues greater than 1 (4.11 and 1.56) and (4.32 and 1.42), but the scale items soc1, soc2 and soc3 formed an uninterpretable second factor. Another CFA, using sample 4, showed acceptable model fit after removing those three items and also soc11 (RMSEA = 0.05; CFI = 0.98; TLI = 0.99; and WRMR = 0.71).

**Conclusion:**

The results indicate that the SOC-13 scale needs further adjustments. The one-factor model with nine items showed a good statistical fit, but the implications of excluding items should be further investigated, considering the scale's content validity, cross-cultural adaptation and theoretical background.

## Background

Promoting population health is a significant goal of the World Health Organization, moving from a disease-centred to a health-centred focus. This approach also emphasises adopting the so-called “salutogenic” model and its construct Sense of Coherence (SOC), which tries to understand how people cope with life adversities and maintain their health [1]. Indeed, it has been shown that the way people deal with stressful events impacts their mental health, obesity, different types of cancer, cardiovascular diseases, and health-related behaviours [2–5]. Consistently, adolescents and their mothers with higher SOC have less dental caries, and higher SOC is associated with greater probability of having preventive dental appointments and less gingival bleeding [6–8]. Higher SOC levels in adolescents were associated with fewer decayed teeth in crude but not adjusted models [6], but a recent systematic review showed a strong effect on dental caries [9]. Higher SOC was also associated with more frequent toothbrushing, lower soda drink consumption, fewer decayed teeth, and fewer oral health-related impacts [10].

The SOC scale proposed by Antonovsky has been widely used in versions of 13 and 29 items [1, 11]. Originally developed in English, it has been translated into 49 languages and used at least in 48 countries [11, 12], indicating some face and content validity. Nonetheless, its factorial structure remains unclear. On the one hand, assuming one overall factor, 124 studies have shown an acceptable Cronbach's alpha ranging from 0.70 to 0.95 for the 29 items version, and 127 studies have shown  values  from 0.70 to 0.92 for the 13 item-SOC scale [12, 13]. On the other hand, authors using factor analysis have reported 2, 3 and up to 5 factors [12, 14–17], suggesting it may be a multidimensional scale. The original three-factor configural model of SOC has been rejected in some studies, and a different arrangement (SOC-R), also with three factors, has been proposed [18–20].

The Brazilian version of the SOC scale used in the present study is an adaptation of the Portuguese version from Portugal for a study carried out among Brazilian adolescent students and their mothers in 1997 [6, 8] and it has been widely used since then. However, it has not been subjected to rigorous factor analysis [21]. Factor analysis is essential to assess internal construct validity because it examines the underlying dimensions of the scale that the items are purported to represent. An exploratory factor analysis of another Brazilian version used in cardiac patients suggested a bad fit for the three-factor model and low loadings in some items, then authors concluded that one factor could be the best factorial solution [22]. Evaluating the factorial structure of the Brazilian version is essential also to assess the cross-cultural adaptation process and may provide further support for future studies in other countries, as the scale is expected to have a similar structure. Thus, the objective of this study was to assess the dimensional structure of the Brazilian version of the Sense of Coherence scale with 13 items according to Antonovsky’s theory.

### The Sense of Coherence scale

Antonovsky’s SOC scale, also named The Orientation to Life Questionnaire, has been used in its full (29-item) and short (13-item) versions [1]. Both measure three dimensions (comprehensibility, manageability, and meaningfulness), which are theoretically distinguishable but interrelated and, at some point, one can overlap each other conforming to one dimension. Antonovsky suggested that “it might seem possible to assign three separate sub-scores” [1] (page 87) as correlations among the three aforementioned dimensions in an Israeli sample were 0.45, 0.59 and 0.62, showing they are only moderately correlated. However, exploratory factor analysis carried out by Antonovsky found that the three components were not empirically separable and then he proposed using an overall score (one dimension). Difficulties in factor analysis to separate items in clear clusters may be due to the fact that Pearson's correlation, used at that time, underestimated the magnitude of correlations making all items similarly low and not able to distinguish different clusters. Currently, it is possible to run factor analysis using an adequate estimator for short ordinal items, that is, a polychoric correlation matrix.

Antonovsky described the three dimensions of the SOC. The first is "comprehensibility” (questions 2, 6, 8, 9 and 11). It is the cognitive component: “the stimuli deriving from onus’s internal and external environments in the course of living are structured, predictable, and explicable”. The second dimension is “manageability” (questions 3, 5, 10 and 13). It is the instrumental component: “the resources are available to one to meet the demands posed by these stimuli". The third dimension is “meaningfulness” (questions 1, 4, 7 and 12), the motivational component: “these demands are challenges, worthy of investment and engagement” [1]. It is considered the most important SOC component. The specific questions of SOC-13 used in this study are summarised in Table 1.

**Table 1:** Sense of Coherence items and their configural structure

Dimension	Number	Item	Options for answers
Meaningfulness	Soc01	“Do you have the feeling that you don't really care about what goes on around you?”	“Very seldom or never” up until “very often”
Comprehensibility	Soc02	"Has it happened in the past that you were surprised by the behaviour of people whom you thought you knew well?”	“never happened” up until “always happened”
Manageability	soc03	“Has it happened that people whom you counted on disappointed you?”	“never happened” up until “always happened”
Meaningfulness	Soc04	“Until now your life has had”	“No clear goals and propose” up until “Very clear and purpose”
Manageability	Soc05	“Do you have the feeling that you're being treated unfairly?”	“Very often” up until "Very seldom or never”
Comprehensibility	Soc06	“Do you have the feeling that you are in an unfamiliar situation and don't know what to do?”	“Very often” up until "Very seldom or never”
Meaningfulness	Soc07	“Doing things you do every day is”	“A source of pleasure and satisfaction” up until “A source of pain and boredom”
Comprehensibility	Soc08	“Do you have very mixed-up feelings and ideas?”	“Very often” up until "Very seldom or never”
Comprehensibility	Soc09	“Does it happen that you have feelings inside you would rather not feel?”	“Very often” up until "Very seldom or never”
Manageability	Soc10	“Many people—even those with a strong character—sometimes feel like sad sacks (losers) in certain situations. How often have you felt this way in the past?”	“Never” up until “Very often”
Comprehensibility	Soc11	“When something happened, have you generally found that”	“You overestimated or underestimated its importance” up until “You saw things in the right proportion”
Meaningfulness	Soc12	“How often do you have the feeling that there's little meaning in the things you do in your daily life?”	“Very often” up until "Very seldom or never”
Manageability	Soc13	“How often do you have feelings that you're not sure you can keep under control?”	“Very often” up until "Very seldom or never”

## Methods

### Data sources and participants

This study is a secondary analysis of four independent cross-sectional Brazilian studies on oral health-related outcomes, using the 13-item SOC scale. The first study (Sample 1) was conducted on 1760 mothers and 1771 adolescents to evaluate the impact of primary health care services in south Brazil in 2011 [23]. The second (Sample 2) comprised 1100 adults to investigate the influence of psychosocial factors on health conditions in south Brazil during 2006–2007 [24]. The third study (Sample 3) investigated the relationship between SOC and oral health in a sample of 720 adults and older individuals in south Brazil during 2008–2009 [25]. The fourth (sample 4) is a study on the association between adolescents' SOC and their oral health in 664 students in mid-west Brazil in 1997 [6]. Datasets 2 to 4 had items as ordered categories from 1 to 7, as proposed in the original version of the scale, while dataset 1 had all SOC items as ordered categories from 1 to 5.

### Data analysis

#### Sample 1—confirmatory factor analysis

Confirmatory factor analysis was conducted to test the dimensional structure of the 13-item SOC scale in two models. The first (M1) was derived from Antonovsky’s postulated theory that the scale was designed to provide a single measure and the analysis consisted of a one-factor model. The second (M2) was based on the three SOC theoretical dimensions (comprehensibility, manageability, and meaningfulness), according to which specific items should load in each factor. A difference between M1 and M2 was tested using DIFFTEST (provided in the Mplus 7.11 software).

We applied the Weighted Least Square Mean and Variance Adjusted (WLSMV) estimator to analyse the SOC scale [26]. The overall goodness-of-fit of each model was evaluated using the comparative parameters provided by the software. Values < 0.05 for Root Mean Square Error of Approximation (RMSEA) and Standardised Root Mean Square Residual (SRMR) suggest close approximate (adequate) fit, whereas values > 0.10 indicate poor fit. The Comparative Fit Index (CFI) and the Tucker–Lewis index (TLI) represent incremental fit, whereas values > 0.95 are indicative of good fit. A Weighted Root Mean Square Residual (WRMR) value < 1.0 indicates a good fit. Internal consistency was assessed using McDonald omega coefficient for each factor separately using standardised items.

#### Samples 2 and 3—exploratory factor analysis

Results of the CFA of Sample 1 showed a poor fit, leading us to reject both models tested. Exploring Sample 1 to obtain a better fit would result in a model far from the postulated theoretically. Then, exploratory factor analysis was carried out in two other datasets to reassess the configural validity, that is, how items allocate when freely estimated. Such a step is considered an initial part of the internal validation process in the factor analysis [27]. Polychoric correlations were used throughout all analyses of all datasets for factor analysis. The extraction method was iterated principal factors. The number of factors was defined using Parallel Analysis (PA) method (paran command in Sata with 1000 replications in a PCA) in combination with the theoretical interpretability of the factors. Furthermore, items were retained in a specific factor if their loadings were > |0.3| [27]. The current analysis used the geomin oblique rotation. Communality measures the common factor variance of an item and it equals 1-uniqueness (δ). A uniqueness of > |0.70| indicates that an item may be unreliable, while a value ≤ |0.70| indicates that the factor accounts for a large percentage of the item variance.

Our next step was to evaluate Bartlett’s test of sphericity and the Kaiser–Meyer–Olkin measure of sampling adequacy. Statistically significant p-values (p < 0.05) in Bartlett's test and measures greater than 0.5 in the Kaiser–Meyer–Olkin test indicated that we could proceed with factor analysis [28]. According to Hair et al. [28], at this stage, items with an adequacy value < 0.50 should be considered for exclusion. Internal consistency was assessed using McDonald omega coefficient for the whole scale using standardised items. All EFA analyses were performed in Stata 16.1 and MPlus version 7.11.

#### Sample 4—confirmatory factor analysis

In this dataset, we conducted the Confirmatory Factor Analysis (CFA) using results from previous EFA in Samples 2 and 3 to propose alternative models. All models departed from 1-factor solution. In Model 1, five residual correlations were added until one acceptable absolute and one relative fit indices were obtained. In Model 2, items with very poor performance were removed (items 1 and 11), and residual correlations were added to obtain an acceptable fit as previously described. In Model 3, highly correlated items (items 2 and 3) were removed, and additional residual correlations were added. In Model 4, items with poor performance (1 and 11) were removed as well as item 2 (highly correlated with item 3). Finally, in Model 5, items with high correlation and poor performance were removed (1, 2, 3 and 11).

Robust weighted least squares mean and variance adjusted (WLSMV) estimator was used [26]. The measurement errors (uniqueness) and loadings were calculated. The goodness-of-fit of the model to the data was evaluated using the ordinary comparative parameters provided by the software. The Root Mean Square Error of Approximation (RMSEA) is an absolute fit index and incorporates a penalty for poor model parsimony [26, 28]. Values lower than 0.05 suggest close approximate (adequate) fit, whereas values equal or above 0.10 indicate poor fit suggesting that the model should be rejected. The Comparative Fit Index (CFI) and the Tucker-Lewis index (TLI) represent incremental fit indices. Both ranges from zero to one, and values > 0.95 indicate a good fit. An overall conclusion about the fit of each model can be obtained by considering these indices simultaneously.

## Results

### Confirmatory factor analysis (sample 1)

The sample was composed of school adolescents between 12 and 19 years of age (mean = 14.1 years and standard deviation ± 2.2) from 36 cities in southern Brazil. Their household income was between BRL 500.00 and 1500.00, with an average per capita income equivalent to BRL 829.27, a value above the minimum wage in the Brazilian state where data were collected. For the sample of mothers, the McDonald omega was 0.77 for the whole scale, and for factors 1, 2 and 3 it was, respectively 0.60, 0.55, and 0.63. For the sample of teenagers, the McDonald omega was 0.66 for the whole scale, and for factors 1, 2 and 3, it was 0.50, 0.49, and 0.54, respectively.

The outcome of the CFA is presented in Table 2 with the one-factor model (M1) and three-factor model (M2) in adolescents and adults separately. The one-factor analysis indicated low item loadings apart from SOC06, SOC08, SOC09 and SOC10 (loading > 0.60) in adults. Several items with low loadings were found in the three-factor model too. The CFA for the total score model did not indicate an acceptable fit (Table 2) neither for the one-factor model in adults (RMSEA = 0.12; CFI = 0.78; TLI = 0.73; and WRMR = 3.28) and nor for the three-factor model (RMSEA = 0.10; CFI = 0.87; TLI = 0.84; and WRMR = 2.50). Results regarding the adolescents showed worse fit for both the one-factor model (RMSEA = 0.10; CFI = 0.70; TLI = 0.64; and WRMR = 2.98) and the three-factor model (RMSEA = 0.09; CFI = 0.79; TLI = 0.74; and WRMR = 2.85). The model showed no satisfactory result and both models were rejected.

**Table 2:** Confirmatory factor analysis of the SOC-13 scale in mothers and their adolescent children from 36 south Brazilian municipalities

Item	Mothers (n = 1718)						Adolescents (n = 1767)					
	M1-1 Factor		M2-3 Factors				M1-1 Factor		M2-3 Factors			
	Factor1		Factor1	Factor2	Factor3		Factor1		Factor1	Factor2	Factor3	
	λ	δ	λ	λ	λ	δ	λ	δ	λ	λ	λ	δ
SOC01	0.26	0.93			0.41	0.84	0.02	1.00			0.30	0.91
SOC02	0.48	0.77	0.49			0.76	0.46	0.79	0.47			0.78
SOC03	0.54	0.71		0.54		0.71	0.51	0.73		0.51		0.74
SOC04	− 0.44	0.80			− 0.65	0.57	− 0.18	0.97			− 0.49	0.76
SOC05	− 0.47	0.78		− 0.46		0.79	− 0.38	0.85		− 0.37		0.87
SOC06	0.67	0.53	0.68			0.54	0.54	0.71	0.53			0.73
SOC07	0.46	0.79			0.63	0.61	0.25	0.94			0.48	0.77
SOC08	0.66	0.57	0.65			0.58	0.58	0.67	0.56			0.68
SOC09	0.60	0.64	0.60			0.64	0.53	0.72	0.53			0.72
SOC10	0.66	0.56		0.64		0.59	0.57	0.68		0.55		0.70
SOC11	− 0.17	0.97	− 0.16			0.98	0.01	1.00	0.01			1.00
SOC12	0.55	0.70			0.69	0.53	0.41	0.83			0.66	0.56
SOC13	0.50	0.75		0.49		0.76	0.47	0.78		0.46		0.79
Correlations F1*				1.14	0.57					1.09	0.38	
Correlations F2*					0.55						0.40	
RMSEA	0.12			0.10			0.10			0.09		
CFI	0.78			0.87			0.70			0.79		
TLI	0.73			0.84			0.64			0.74		
WRMR	3.28			2.50			2.90			2.85		
*ΔM1–M2 DIFFTEST*	*p* < 0.01						*p* < 0.01					

Factor 1: Comprehensability, Factor 2: Manageability, Factor 3: Meaningfulness

λ = loadings; δ = uniqueness; *Factor score correlation; RMSEA = Root Mean Square Error Of Approximation; CFI = comparative fit index; TLI = Tucker-Lewis index; WRMR = Weighted Root Mean Square Residual

### Exploratory factor analysis (sample 2)

This is a sample from Sao Leopoldo city in southern Brazil and consisted of 1098 individuals. The study population consisted mainly of women (71.8%), white people (84%), aged between 20 and 49 years (mean = 44.3 years and standard deviation ± 15.8), and with 5–11 years of schooling (65.2%). The adjusted Eigenvalues from PA were 4.11, 1.56 and 0.91. Although two components were recommended to be retained, we extracted three models with 1, 2 and 3 factors to assess how items behave in different situations and because theoretically the scale should have three factors. They explained 72.4% of the shared variance and the addition of a third factor increased it to 79.2%. For this sample, the McDonald omega was 0.78 for the whole scale.

Based on the theory that SOC could have three factors, we extracted loadings up to three factors in the exploratory analysis using Mplus. The fit of the one-factor model showed unacceptable values: RMSEA = 0.17, CFI = 0.70, TLI = 0.64. The fit of the two-factor model showed acceptable values: RMSEA = 0.07, CFI = 0.97, TLI = 0.95; however, the first factor was not interpretable. The fit of the three-factor model showed acceptable values: RMSEA = 0.05, CFI = 0.98, TLI = 0.97 but the first and the third factors were not interpretable (Table 3).

**Table 3:** Exploratory factor analysis of the SOC-13 scale in adults and elderly of two south Brazilian municipalities.

Variable	Sample 2 (São Leopoldo, n = 1098)						Sample 3 (Porto Alegre, n = 720)					
	One Factor Solution	Two-factor solution		Three factorsolution			One Factor Solution	Two-factor solution		Three-factor solution		
	Factor1	Factor1	Factor2	Factor1	Factor2	Factor3	Factor1	Factor1	Factor2	Factor1	Factor2	Factor3
	λ	λ	λ	λ	λ	λ	λ	λ	λ	λ	λ	λ
SOC01	0.34	− 0.01	0.37	0.03	***0.41***	− 0.05	0.19	0.04	0.17	0.15	0.00	0.27
SOC02	***0.63***	***0.84***	− 0.02	***0.81***	− 0.04	0.02	***0.52***	***0.77***	− 0.01	***0.83***	0.00	0.13
SOC03	***0.66***	***0.85***	0.03	***0.91***	0.02	− 0.05	***0.61***	***0.76***	0.12	***0.68***	0.24	− 0.01
SOC04	***0.45***	− 0.20	***0.60***	− 0.17	***0.63***	− 0.03	0.26	− 0.29	***0.47***	− 0.15	0.20	0.35
SOC05	***0.43***	0.11	***0.40***	0.13	***0.42***	− 0.01	***0.58***	0.39	0.35	0.34	***0.43***	− 0.02
SOC06	***0.66***	0.02	***0.70***	0.02	***0.63***	0.12	***0.66***	0.14	***0.60***	0.10	***0.62***	0.02
SOC07	***0.49***	− 0.04	***0.55***	− 0.02	***0.56***	0.01	***0.56***	− 0.10	***0.65***	− 0.02	***0.48***	0.24
SOC08	***0.71***	0.09	***0.71***	0.02	***0.45***	***0.41***	***0.75***	0.01	***0.78***	0.02	***0.72***	0.11
SOC09	***0.56***	0.15	***0.53***	− 0.01	0.00	***0.87***	***0.70***	0.19	***0.61***	0.00	***0.88***	− 0.32
SOC10	***0.48***	0.24	***0.40***	0.22	0.28	0.18	***0.60***	0.23	***0.48***	0.20	***0.52***	0.01
SOC11	0.38	0.04	0.39	0.03	0.31	0.13	***0.31***	− 0.02	0.34	0.04	0.23	0.16
SOC12	***0.62***	− 0.06	***0.69***	− 0.03	***0.69***	0.03	***0.57***	− 0.09	***0.66***	0.01	***0.47***	0.29
SOC13	***0.62***	− 0.02	***0.67***	− 0.04	***0.54***	0.23	***0.63***	0.00	***0.66***	− 0.05	***0.68***	− 0.01
Model Fit Indices												
RMSEA	0.17	0.07		0.05			0.12	0.04		0.04		
CFI	0.70	0.97		0.98			0.84	0.98		0.99		
TLI	0.64	0.95		0.97			0.80	0.98		0.98		
SRMR	0.12	0.04		0.03			0.08	0.03		0.02		

The bold italics refer to loading values >|0.30|

λ = loadings; RMSEA = Root Mean Square Error Of Approximation; CFI = comparative fit index; TLI = Tucker-Lewis index; WRMR = Weighted Root

### Exploratory factor analysis (sample 3)

This is a sample from Porto Alegre, a capital city in southern Brazil and consisted of 720 individuals. The age range of the participants was 50–74 years (mean = 60.2 years and standard deviation ± 7.5), and they were predominantly female (57.8%), 26.2% earned two minimal wages or less monthly, and 29.8% had less than six years of study. The polychoric correlation matrix shows inter-item correlations used for exploratory factor analysis. The adjusted Eigenvalues from PA were 4.32, 1.42 and 0.86. Although two components were recommended to be retained, we extracted three models with 1, 2 and 3 factors to assess how items behave in different situation and because theoretically the scale should have three factors. They explained 74.0% of the shared variance and the addition of a third factor increased it to 79.6%. For this sample, the McDonald omega was 0.81 for the whole scale.

As in the previous sample, factors loadings we extracted up to 3 factors in exploratory analysis using Mplus. The fit of the one-factor model showed unacceptable values: RMSEA = 0.12, CFI = 0.84, TLI = 0.80 The fit of the two-factor model showed acceptable values: RMSEA = 0.04, CFI = 0.98, TLI = 0.98; however, the first factor was not interpretable. The fit of the three-factor model showed acceptable values: RMSEA = 0.04, CFI = 0.99, TLI = 0.98 but the first and the third factors were also not interpretable. The one-factor model, SOC01, SOC04 and SOC11, showed a low loading (< 0.40). The model with two and three factors showed a good fit, but the results differed from the theory, and the factors were uninterpretable (Table 3).

### Confirmatory factor analysis (sample 4)

This is a sample from Goiania city in Middle-West Brazil. It consisted of 664 adolescents, all 15 years old (344 female and 320 male), whose mothers were predominantly from low socioeconomic status (60.7%) and had low levels of schooling (36.8%). For the sample of adolescents, the McDonald omega was 0.78 for the whole scale (one factor).

Based on previous results from studies 2 and 3, we decided to exclude some items and test five different one-factor models using CFA. The results indicated high item loadings (Table 4) except for the item SOC01 and SOC11 (loading < 0.40). All alternative models yielded good fit indices, but the best fit was obtained for a model without 4 items (SOC01, SOC02, SOC03 and SOC11) with RMSEA = 0.05, CFI = 0.98, TLI = 0.97 and WRMR = 0.71 (Table 4).

**Table 4:** Standardized coefficients from confirmatory factor analysis of the SOC-13 scale among adolescents in one capital city of Middle-West Brazil (N = 664)

Variable	Initial model		Model 1		Model 2		Model 3		Model 4		Model 5	
	λ	δ	λ	δ	λ	δ	λ	δ	λ	δ	λ	δ
SOC01	*0.02*	1.00	*0.01*	1.00			*0.03*	1.00				
SOC02	**0.46**	0.79	*0.37*	0.86	*0.37*	0.86			*0.37*	0.87		
SOC03	**0.47**	0.78	*0.36*	0.87	*0.39*	0.85						
SOC04	**0.48**	0.77	**0.50**	0.75	**0.50**	0.75	**0.52**	0.73	**0.51**	0.74	**0.52**	0.73
SOC05	**0.58**	0.66	**0.57**	0.67	**0.57**	0.68	**0.60**	0.65	**0.60**	0.64	**0.61**	0.63
SOC06	**0.64**	0.60	**0.67**	0.56	**0.67**	0.55	**0.65**	0.58	**0.67**	0.55	**0.68**	0.55
SOC07	**0.57**	0.68	**0.56**	0.68	**0.55**	0.70	**0.61**	0.63	**0.60**	0.64	**0.61**	0.63
SOC08	**0.63**	0.61	**0.61**	0.63	**0.61**	0.63	**0.64**	0.59	**0.60**	0.64	**0.59**	0.65
SOC09	**0.61**	0.63	**0.53**	0.72	**0.55**	0.70	**0.55**	0.70	**0.52**	0.73	**0.49**	0.76
SOC10	**0.50**	0.75	**0.52**	0.73	**0.51**	0.74	**0.48**	0.77	**0.50**	0.75	**0.49**	0.76
SOC11	*0.34*	0.88	*0.34*	0.88			*0.36*	0.87				
SOC12	**0.55**	0.70	**0.57**	0.67	**0.57**	0.67	**0.58**	0.67	**0.58**	0.67	**0.59**	0.66
SOC13	**0.56**	0.69	**0.53**	0.72	**0.52**	0.73	**0.50**	0.75	**0.50**	0.75	**0.49**	0.76
*Residual correlations*												
SOC02 WITH SOC03			0.45	0.44								
SOC03 WITH SOC09			0.16									
SOC05 WITH SOC07			0.18		0.19							
SOC08 WITH SOC09			0.21		0.20				0.23		0.25	
SOC09 WITH SOC13			0.25		0.24		0.28		0.27		0.29	
*Model fit indices*												
RMSEA	0.11		0.06		0.07		0.07		0.07		0.05	
WRMR	1.60		0.96		0.97		0.94		0.91		0.71	
CFI	0.84		0.95		0.95		0.95		0.96		0.98	
TLI	0.81		0.94		0.94		0.94		0.94		0.97	

The bold and italics values refers to loading values >|0.30|

λ = loadings; δ = uniqueness; RMSEA = Root Mean Square Error Of Approximation; CFI = comparative fit index; TLI = Tucker-Lewis index; WRMR = Weighted Root Mean Square Residual;

## Discussion

The present study investigated the configural structure of the Brazilian version of the SOC-13 used in oral health studies and we could not confirm the initial hypothesis that either one or three theoretical dimensions would have an adequate fit. To the best of our knowledge, this is the most comprehensive study on the configural validity of SOC-13, at least in Brazil. A modified model, removing four items (SOC01, SOC02, SOC03 and SOC11), showed that the one-factor model had a more parsimonious structure with acceptable fit indices. Other alternative models yielded an adequate fit when several additional residual correlations were implemented.

One of the assumptions in this study was that the SOC-13 had a cross-cultural equivalence for the Brazilian population [21], but some items had very low loading in one-factor model and were removed to achieve an acceptable fit. In line with our study, an acceptable fit was achieved in the Norwegian version only after dropping items SOC02 to SOC04 and SOC11 [13]. A systematic review on cross-cultural adaptation to Brazil reported that individuals had difficulties in understanding items SOC01, SOC06 and SOC11 [21]. Despite a consensus in the literature regarding the face and content validity of the scale, we observed that researchers from other countries also pointed out similar psychometric problems concerning those items and a factorial structure different from the originally proposed, suggesting that some items may be culturally sensitive. International studies reported that some items loaded in different factors than those theorised [16, 29]; therefore, rejecting the original three-factor model. Interviewees encountered difficulties in answering and interpreting SOC02 and SOC03 and considered those items as addressing the same question [15]. This may explain why SOC02 and SOC03 were reported to have a higher residual correlation than any other pair of items in the scale [16, 30, 31], forming a factor on their own.

Our final CFA supports a modified one-factor model after removing four items. Differently, a previous study, performing CFA on SOC-29 [32], showed that the three-dimension model presented a better fit than the one dimension [33]. Konttinen argued that SOC-13 should have three distinct dimensions [34], but this and other studies have also failed to confirm those dimensions [13, 15–17, 34]. Notably, a short instrument (13 items) may not be able to capture three distinct factors, and this fact can be relevant if dimensions are theoretically correlated and items have cross-loadings. Not surprisingly, the three conceptual dimensions have never been reported in previous confirmatory factor analyses of the SOC-13 scale [12, 35].

The first version of the Sense of Coherence scale consisted of 29 items, and an abridged version with 13 items was later developed by Antonovsky [1, 35]. However, methodological details on the development of this version have not been identified in the literature. If a golden standard is not available, the best approach to shorten a scale is to combine an expert-based approach with statistical techniques [36]. In addition, for multidimensional scales, each sub-scale should be subject to a specific shortening process to ensure that it will keep the same dimensional structure. An abridged version that mixed up dimensions may be less valuable than the original scale, but it became popular because it is helpful in large surveys to tap an overall dimension.

This study has some strengths and limitations. Although we have four large samples from different parts of Brazil, results can only be generalised with caution. Nonetheless, our findings were similar to other countries with different cultural backgrounds [16, 29–31]. Importantly, our four databases were large, with sample sizes between 664 and 1767 individuals, reducing the chances of random error regarding items loading and other psychometric issues [37]. We were also able to test the configural model of the SOC-13 based on a priori specification of two models for CFA and, after rejecting them, we could explore modifications using EFA in two datasets of different populations. However, removing items is a rather simplistic approach to solving the problem of a statistical misfit as there is growing evidence to suggest that the use of fit indices to define the number of factors and other issues is problematic in many ways [38]. We believe definite changes should be guided by theory and discussed among scholars.

In conclusion, our results indicate that the Brazilian version of SOC-13 scale needs further adjustments. Several alternative models yielded a good fit, after modification in key issues. The one-factor model with nine items showed a good statistical fit and was the most parsimonious, but such changes need further discussion before being implemented in future studies and the theoretical background has to be considered as proposed by Antonovsky [1]. For example, removing items may affect the content validity [39]; thus, qualitative studies with respondents and experts are essential to warrant the theoretical validity of the cross-cultural adaptation process and possibly the shortening process from SOC-29 may be revised.

## Data Availability

Datasets analysed during the current study are not publicly available but are available from the corresponding author on reasonable request.

## Abbreviations

EFA: Exploratory Factor Analysis
CFA: Confirmatory Factor Analysis
SOC: Sense of Coherence
λ: Loadings
δ: Uniqueness
CFI: Comparative fit index
TLI: Tucker-Lewis index
RMSEA: Root Mean Square Error Of Approximation
WRMR: Weighted Root Mean Square Residual
WLSMV: Weighted Least Square Mean and Variance Adjusted

## References

[1] Antonovsky A (1987). Unraveling the mystery of health: how people manage stress and stay well.

[2] Reers H, Jacot A, Forstmeier W (2011). Do zebra finch parents fail to recognise their own offspring?. PLoS ONE.

[3] Drageset J, Eide GE, Hauge S (2016). Symptoms of depression, sadness and sense of coherence (coping) among cognitively intact older people with cancer living in nursing homes-a mixed-methods study. PeerJ.

[4] Nilsson B, Holmgren L, Stegmayr B, Westman G (2003). Sense of coherence–stability over time and relation to health, disease, and psychosocial changes in a general population: a longitudinal study. Scand J Public Health.

[5] Suresky MJ, Zauszniewski JA, Bekhet AK (2008). Sense of coherence and quality of life in women family members of the seriously mentally ill. Issues Ment Health Nurs.

[6] Freire MCM, Sheiham A, Hardy R (2001). Adolescents’ sense of coherence, oral health status, and oral health-related behaviours. Community Dent Oral Epidemiol.

[7] Bonanato K, Paiva SM, Pordeus IA, Ramos-Jorge ML, Barbabela D, Allison PJ (2009). Relationship between mothers’ sense of coherence and oral health status of preschool children. Caries Res Caries Res.

[8] Freire MCM, Hardy R, Sheiham A (2002). Mothers’ sense of coherence and their adolescent children’s oral health status and behaviours. Community Dent Heal.

[9] Torres TAP, Corradi-Dias L, Oliveira PD, Martins CC, Paiva SM, Pordeus IA (2020). Association between sense of coherence and dental caries: systematic review and meta-analysis. Health Promot Int.

[10] Jönsson B, Holde GE, Baker SR (2020). The role of psychosocial factors and treatment need in dental service use and oral health among adults in Norway. Community Dent Oral Epidemiol.

[11] Mittelmark MB, Sagy S, Eriksson M, Bauer GF, Pelikan JM, Lindström B (2016). The handbook of salutogenesis. Handb. Salut.

[12] Eriksson M, Lindström B (2005). Validity of Antonovsky’s sense of coherence scale: a systematic review. J Epidemiol Community Health.

[13] Klepp OM, Mastekaasa A, Sørensen T, Sandanger I, Kleiner R (2007). Structure analysis of Antonovsky’s sense of coherence from an epidemiological mental health survey with a brief nine-item sense of coherence scale. Int J Methods Psychiatr Res.

[14] von Bothmer MIK, Fridlund B (2003). Self-rated health among university students in relation to sense of coherence and other personality traits. Scand J Caring Sci.

[15] Naaldenberg J, Tobi H, van den Esker F, Vaandrager L (2011). Psychometric properties of the OLQ-13 scale to measure Sense of Coherence in a community-dwelling older population. Health Qual Life Outcomes.

[16] Bernabé E, Tsakos G, Watt RG, Suominen-Taipale AL, Uutela A, Vahtera J (2009). Structure of the sense of coherence scale in a nationally representative sample: the Finnish Health 2000 survey. Qual Life Res.

[17] Söderhamn U, Sundsli K, Cliffordson C, Dale B (2015). Psychometric properties of Antonovsky’s 29-item Sense of Coherence scale in research on older home-dwelling Norwegians. Scand J Public Health.

[18] del C Vega Martínez M, Frías Osuna A, Del Pino Casado R (2019). Validity and reliability of the sense of coherence scale among nursing undergraduate students from a Spanish university. Gac Sanit.

[19] Thoma MV, Mc Gee SL, Fegert JM, Glaesmer H, Brähler E, Maercker A (2018). Evaluation of the revised sense of coherence scale in a representative German sample. PLoS ONE.

[20] Mc Gee SL, Höltge J, Maercker A, Thoma MV (2018). Evaluation of the revised Sense of Coherence scale in a sample of older adults: A means to assess resilience aspects. Aging Ment Health Aging Ment Health.

[21] Scalco GPC, Abegg CC, Celeste RK (2020). Evaluation of the cross-cultural adaptation of Brazilian version of the Sense of Coherence Scale: a systematic review. Cad Saúde Coletiva.

[22] Spadoti Dantas RA, Silva FS, Ciol MA (2014). Psychometric properties of the Brazilian Portuguese versions of the 29- and 13-item scales of the Antonovsky’s Sense of Coherence (SOC-29 and SOC-13) evaluated in Brazilian cardiac patients. J Clin Nurs.

[23] Ely HC, Abegg C, Celeste RK, Pattussi MP (2016). Impact of oral health teams of the Family Health Strategy on the oral health of adolescents in the south of Brazil. Cien Saude Colet.

[24] Ferreira DC, Gonçalves TR, Celeste RK, Olinto MTA, Pattussi MP (2020). Psychosocial aspects and the impact of oral health on quality of life of Brazilian adults. Rev Bras Epidemiol Rev Bras Epidemiol.

[25] Davoglio RS, Fontanive VN, de Oliveira MMC, Abegg C (2020). Sense of coherence and impact of oral health on quality of life in adults and elderly in Southern Brazil. Cien Saude Colet Cien Saude Colet.

[26] Byrne BM (2012). Structural equation modeling with Mplus: basic concepts, applications, and programming.

[27] Kline P (1994). An easy guide to factor analysis.

[28] Hair JF, Black WC, Babin BJ, Anderson RE (2009). Multivariate data analysis.

[29] Lizarbe-Chocarro M, Guillén-Grima F, Aguinaga-Ontoso I, Canga AN (2016). Validation of Antonovsky Orientation to Life Questionnaire (OLQ-13) in a sample of university students in Navarre. An Sist Sanit Navar.

[30] Feldt T, Leskinen E, Kinnunen U, Mauno S (2000). Longitudinal factor analysis models in the assessment of the stability of sense of coherence. Pers Individ.

[31] Hittner JB (2007). Factorial invariance of the 13-item Sense of Coherence scale across gender. J Health Psychol.

[32] Bonacchi A, Miccinesi G, Galli S, Chiesi F, Martire M, Guazzini M (2012). The dimensionality of Antonovsky’s sense of coherence scales. An investigation with Italian samples. TPM Test Psychom Methodol Appl Psychol.

[33] Mautner E, Ashida C, Greimel E, Lang U, Kolman C, Alton D (2014). Are there differences in the health outcomes of mothers in europe and east-asia? A cross-cultural health survey. Biomed Res Int.

[34] Konttinen H, Haukkala A, Uutela A (2008). Comparing sense of coherence, depressive symptoms and anxiety, and their relationships with health in a population-based study. Soc Sci Med.

[35] Antonovsky A (1993). The structure and properties of the sense of coherence scale. Soc Sci Med.

[36] Coste J, Guillemin F, Pouchot J, Fermanian J (1997). Methodological approaches to shortening composite measurement scales. J Clin Epidemiol.

[37] Costello AB, Osborne JW (2005). Best practices in exploratory factor analysis: four recommendations for getting the most from your analysis. Pract Assess Res Eval.

[38] Montoya AK, Edwards MC (2021). The poor fit of model fit for selecting number of factors in exploratory factor analysis for scale evaluation. Educ Psychol Measur.

[39] Ullman JB (2006). Structural equation modeling: reviewing the basics and moving forward. J Pers Assess.

